# Supplementary material for: RNA-Seq Analysis Demystify the Pathways of UV-A Supplementation in Different Photoperiods Integrated with Blue and Red Light on Morphology and Phytochemical Profile of Kale
Source: Antioxidants (Basel). 2023 Mar 16;12(3):737. doi: 10.3390/antiox12030737 (PMC10045344; doi:10.3390/antiox12030737)
Supplement: Supplementary file 1 [file antioxidants-12-00737-s001.zip › antioxidants-2216518-supplementary.pdf]

Table S1. Primers of 15 DEGs

Gene	Accession Number	Forward Primer (5'-3')	Reverse Primer (5'-3')
ACT	LOC106312299	TGGTTGGGATGGGACAGAAG	AATGCCGTGCTCAATAGGGT
ncbi_106295500	LOC106295500	ATCGGTTACCTCGACATT	TCACAGCAGGATCATTACA
ncbi_106300718	LOC106300718	TCAAAGGGAATCAGCAAA	TTACCGCTCAAGAAACCA
ncbi_106296668	LOC106296668	AATTCTACCACGACCTCA	CCACCGTATTCATCTTCTA
ncbi_106318388	LOC106318388	CTTCTTACCGTGGCTTCA	TACCCATCTCATCCTTTCTT
ncbi_106328411	LOC106328411	AAACGAAGCATCCCAAGA	TGAGCCATTCCCAGAGTA
ncbi_106311920	LOC106311920	AACAATCGGACAAGTCGC	CCATAACCCAATCACTGCTA
ncbi_106308421	LOC106308421	GAGTGCCCTGGTGTTGTC	GTGGTCGGGAAGGTAAGA
ncbi_106342634	LOC106342634	TTCTCCCAATCTCACCAA	GAACTCCCAACCTTCCCT
ncbi_106295962	LOC106295962	TGGCGTGGGACAAATAAA	TGGAGTGCCTGAGGAAGG
ncbi_106322682	LOC106322682	TTACAGAGGGGTGAGACAGA	AACCACGCATCCTAAAAGCA
ncbi_106321330	LOC106321330	TTCTATGGAACCCACAACGA	AGACATCAGGACAGGACGAC
ncbi_106318387	LOC106318387	TCCTTTCACTGCTTCTTACC	CACCCATCTCATCCTTTCTT
ncbi_106307722	LOC106307722	ATCTGAAGGAGAACGGTAGC	GGAGGATGAGGAAGAAAAAC
ncbi_106331458	LOC106331458	AGAGTCGTCCTCGCCCTTCA	TTGGTTCCTTGGCCTTCGTA
ncbi_106309520	LOC106309520	CTCCTTCTTTCCCATCTCAT	GGCTTTCTTCATTTCGTTTC

Table S2. Summary of sequencing reads after filtering.

Sample	Raw reads	Clean reads	Mapped reads	Clean bases	Q20	Q30	GC(%)
CK-1	47,163,946	47,083,950	91.01%	7,027,200,604	97.72%	93.23%	47.73%
CK-2	46,732,296	46,648,346	91.20%	6,963,869,670	97.94%	93.80%	47.78%
CK-3	39,426,234	39,356,652	91.26%	5,880,297,602	97.83%	93.51%	47.82%
LS-1	49,853,254	49,763,488	91.21%	7,429,483,537	97.82%	93.48%	47.79%
LS-2	41,652,914	41,579,822	91.40%	6,206,694,348	97.96%	93.86%	47.77%
LS-3	42,222,764	42,149,418	91.78%	6,289,724,527	98.20%	94.45%	47.64%
DS-1	35,736,340	35,668,084	90.90%	5,326,895,587	97.87%	93.61%	47.59%
DS-2	40,381,950	40,311,228	91.17%	6,018,110,169	98.10%	94.21%	47.54%
DS-3	54,259,602	54,160,188	90.62%	8,081,888,288	97.62%	93.01%	47.57%
LDS-1	41,642,550	41,553,578	91.46%	6,202,801,083	97.75%	93.29%	47.97%
LDS-2	40,914,856	40,840,248	91.23%	6,098,610,869	98.04%	94.03%	47.74%
LDS-3	41,000,918	40,925,494	90.74%	6,110,689,061	97.85%	93.59%	47.54%

[Figure S1]

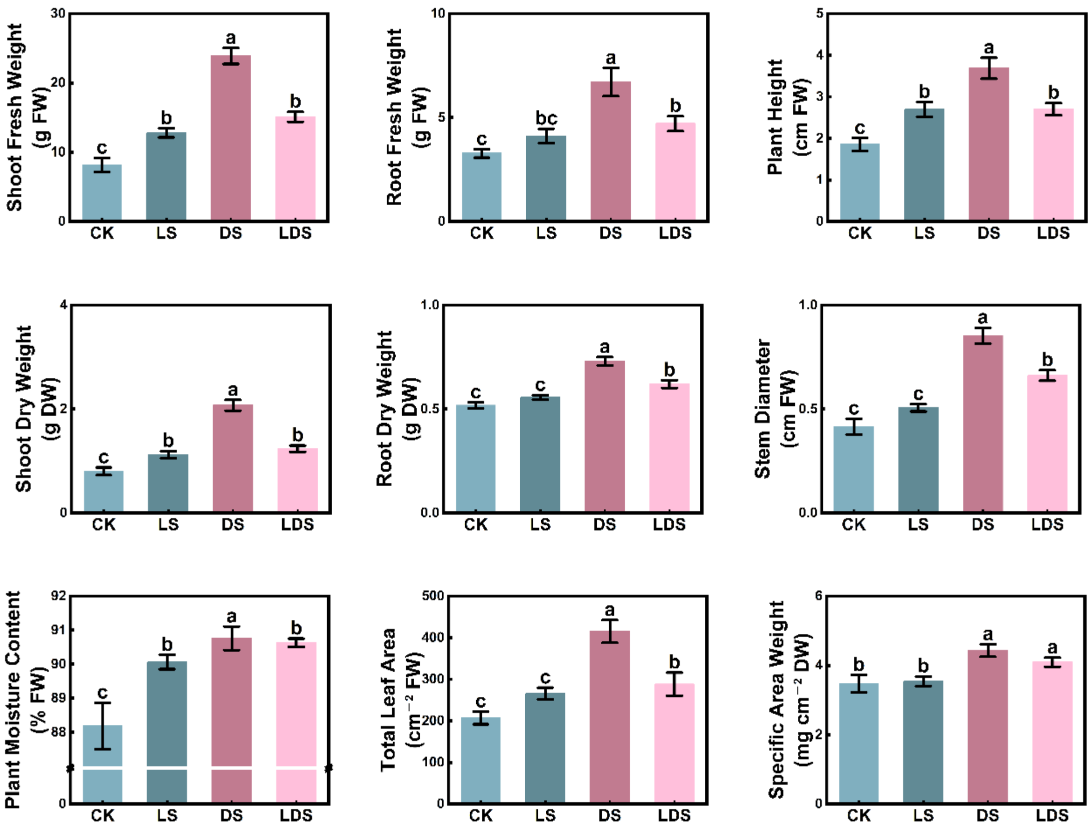

Figure S1. the morphology and biomass of kale under different UV-A treatments. Different lowercase letters reveal significant differences ($p < 0.05$), according to Duncan's test. Vertical bars indicate the standard margin of error. "LS" = Light-UVA Supplementation, "DS" = Dark-UVA Supplementation, "LDS" = Light/Dark-UVA Supplementation

[Figure S2]

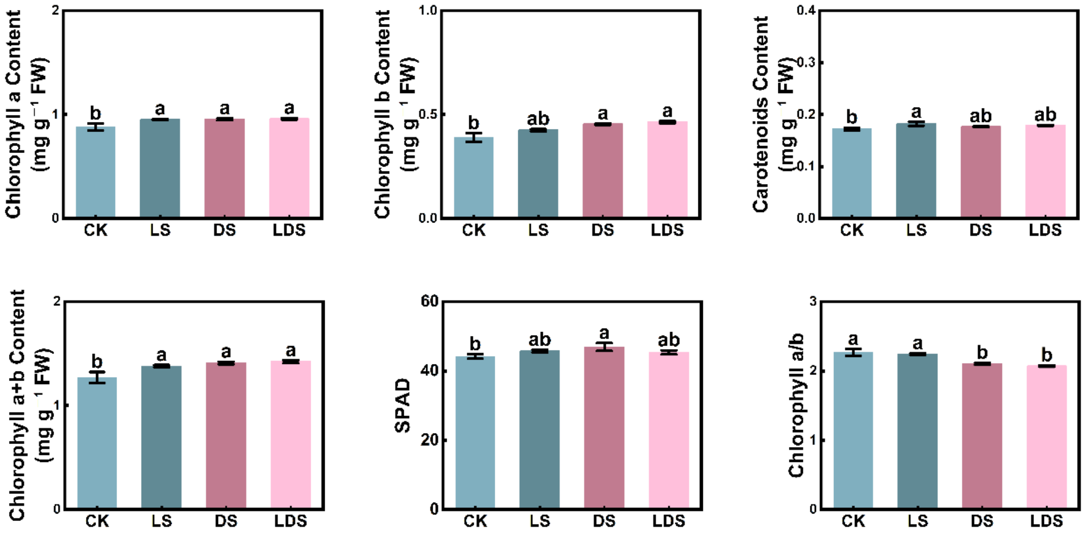

Figure S2. the pigment content of kale under different UV-A treatments. Different lowercase letters reveal significant differences ($p < 0.05$), according to Duncan's test. Vertical bars indicate the standard margin of error. "LS" = Light-UVA Supplementation, "DS" = Dark-UVA Supplementation, "LDS" = Light/Dark-UVA Supplementation

[Figure S3]

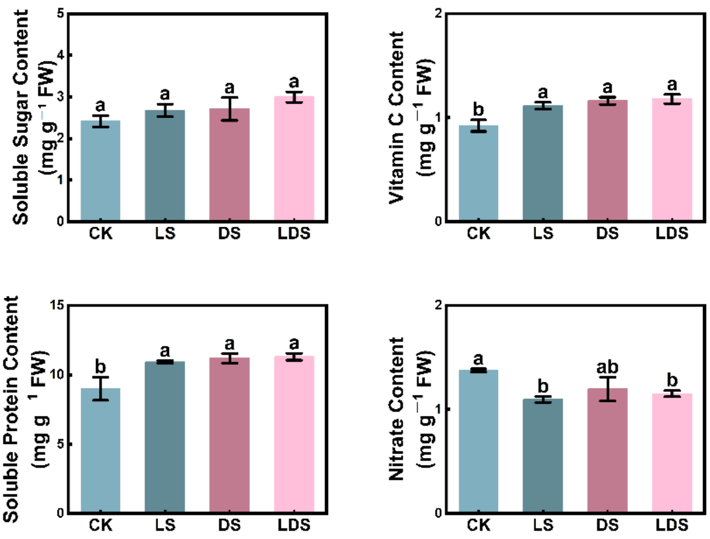

Figure S3. the nutritional quality content of kale under different UV-A treatments. Different lowercase letters reveal significant differences ($p < 0.05$), according to Duncan's test. Vertical bars indicate the standard margin of error. "LS" = Light-UVA Supplementation, "DS" = Dark-UVA Supplementation, "LDS" = Light/Dark-UVA Supplementation

[Figure S4]

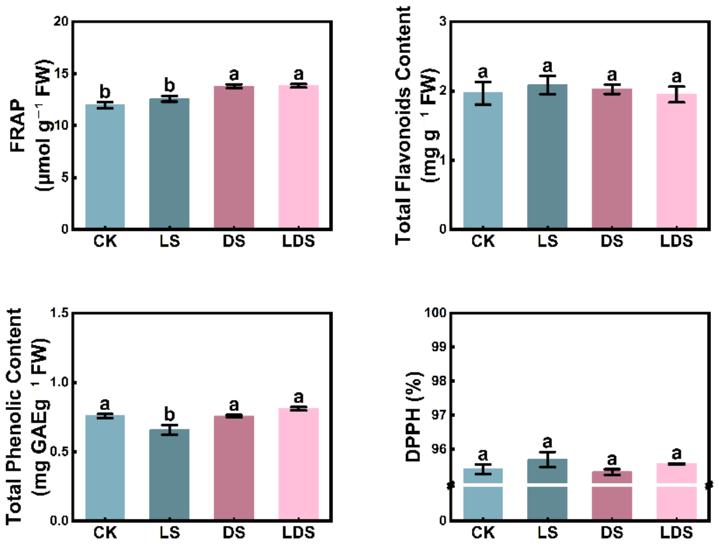

Figure S4. the antioxidant capacity and compounds of kale under different UV-A treatments. Different lowercase letters reveal significant differences ($p < 0.05$), according to Duncan's test. Vertical bars indicate the standard margin of error. "LS" = Light-UVA Supplementation, "DS" = Dark-UVA Supplementation, "LDS" = Light/Dark-UVA Supplementation

[Figure S5]

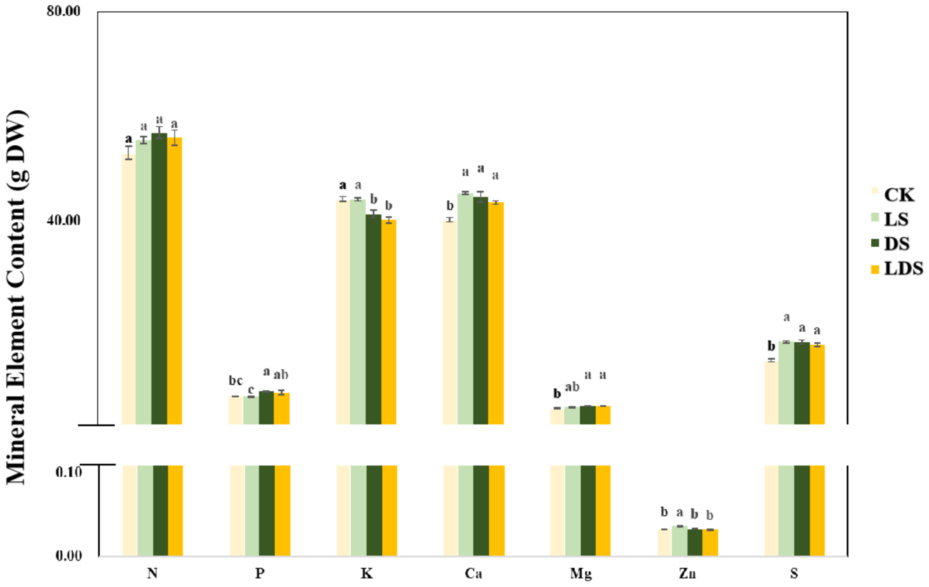

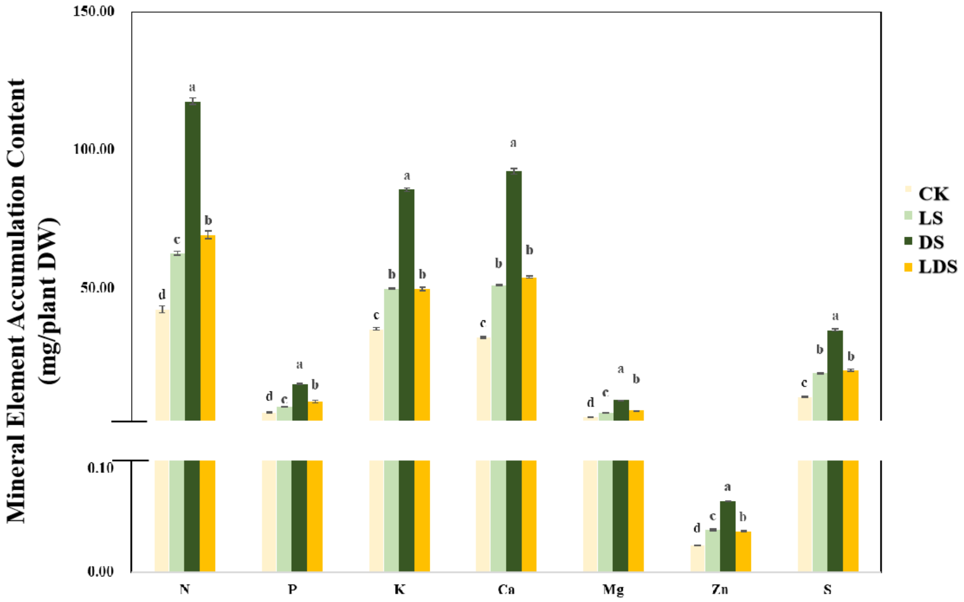

Figure S5. Mineral element and mineral element accumulation contents of kale under different UV-A treatments. Different lowercase letters reveal significant differences (p< 0.05), according to Duncan's test. Vertical bars indicate the standard margin of error. "LS" = Light-UVA Supplementation, "DS" = Dark-UVA Supplementation, "LDS" = Light/Dark-UVA Supplementation

[Figure S6]

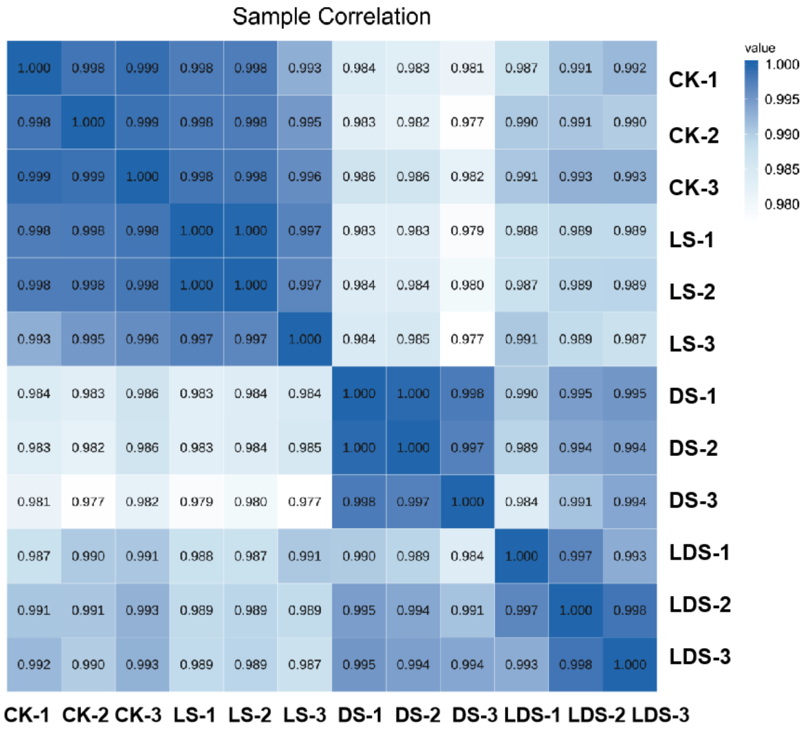

Figure S6. Pearson correlation coefficients of 12 datasets.

[Figure S7]

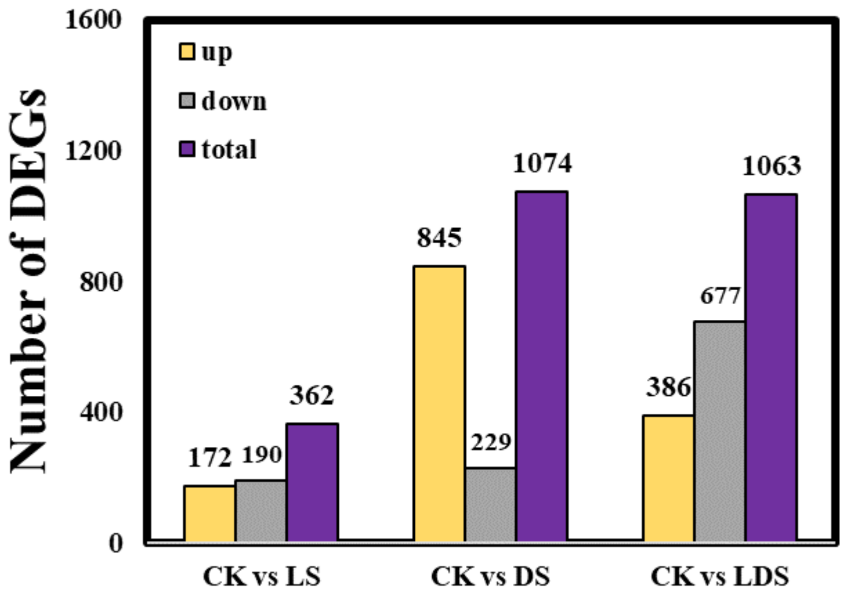

Figure S7. The DEGs among different comparison groups.

[Figure S8]

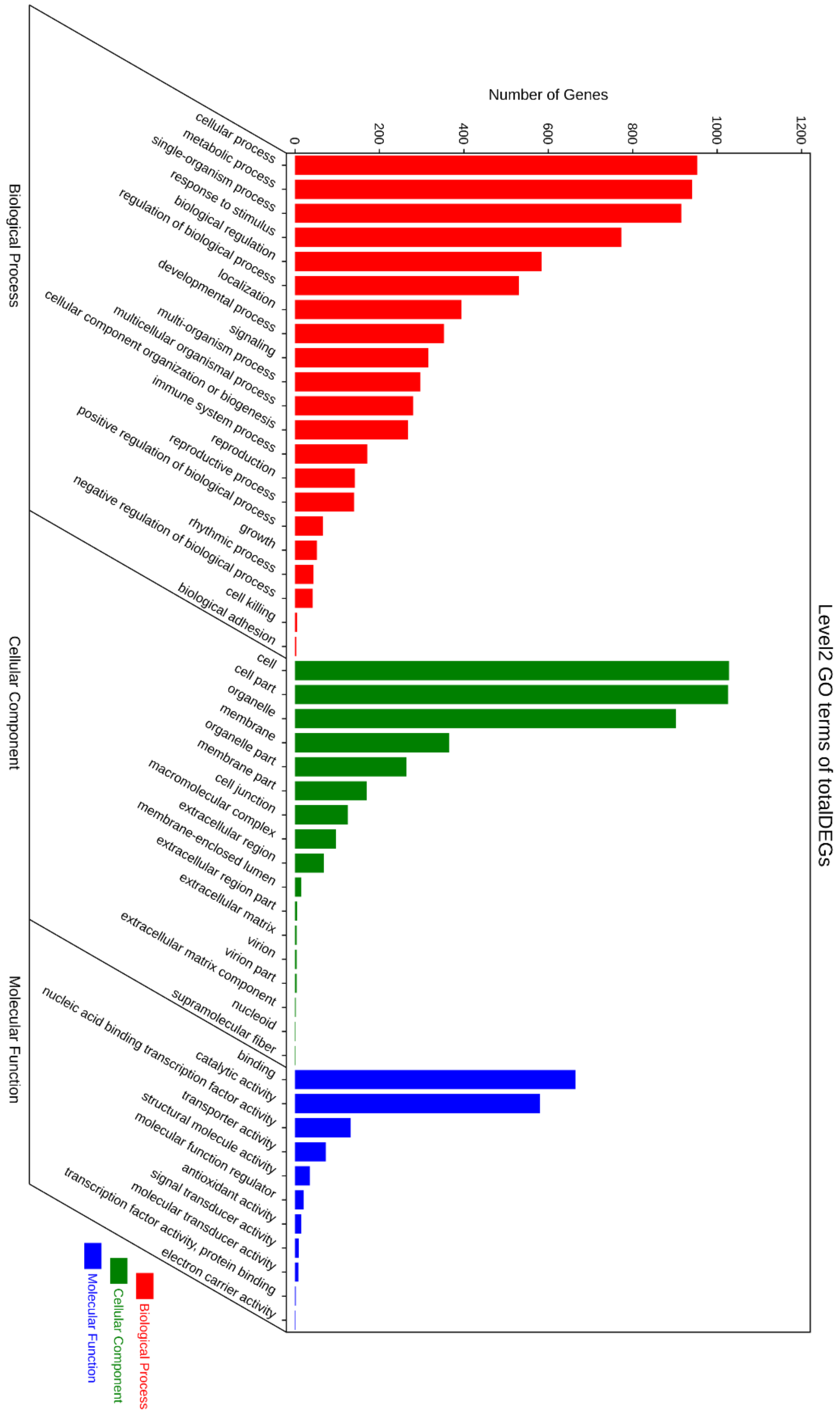

Figure S8. GO enrichment of 2047 DEGs.

[Figure S9]

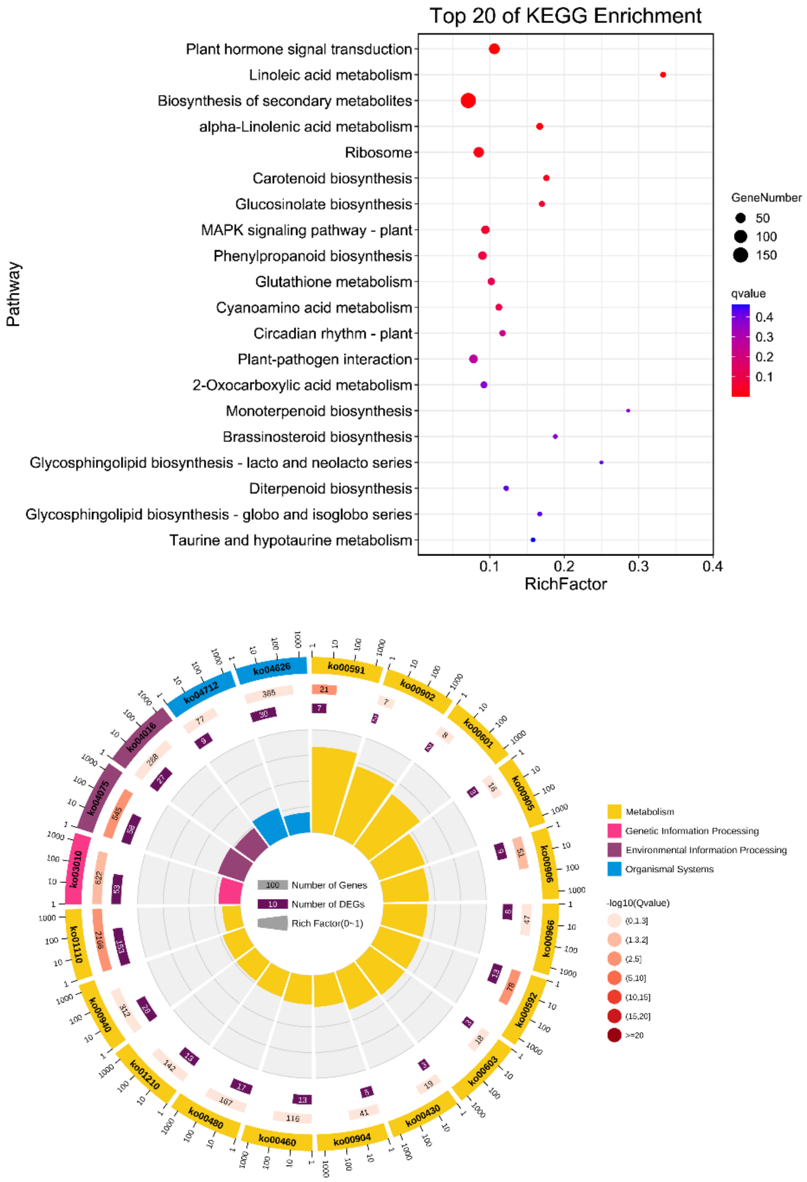

Figure S9. Top 20 KEGG pathways enrichment of 2047 DEGs.

[Figure S10]

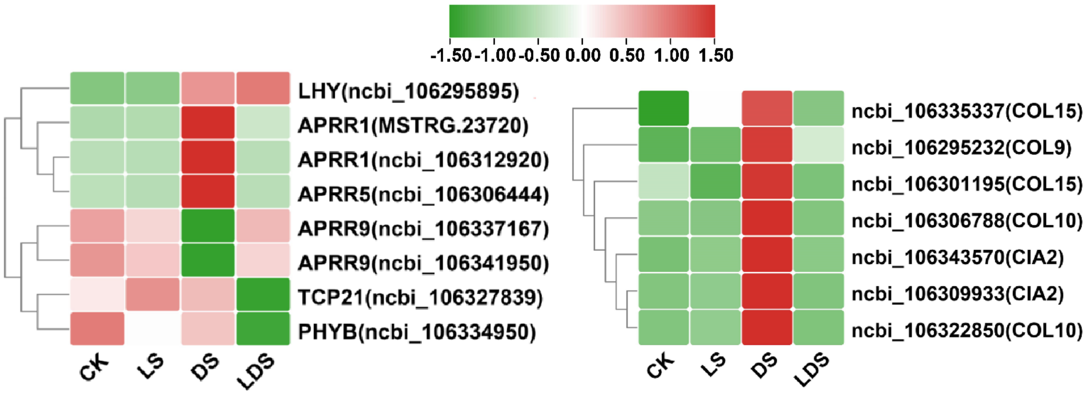

Figure S10. Expression of DEGs related to circadian rhythm – plant pathway.

[Figure S11]

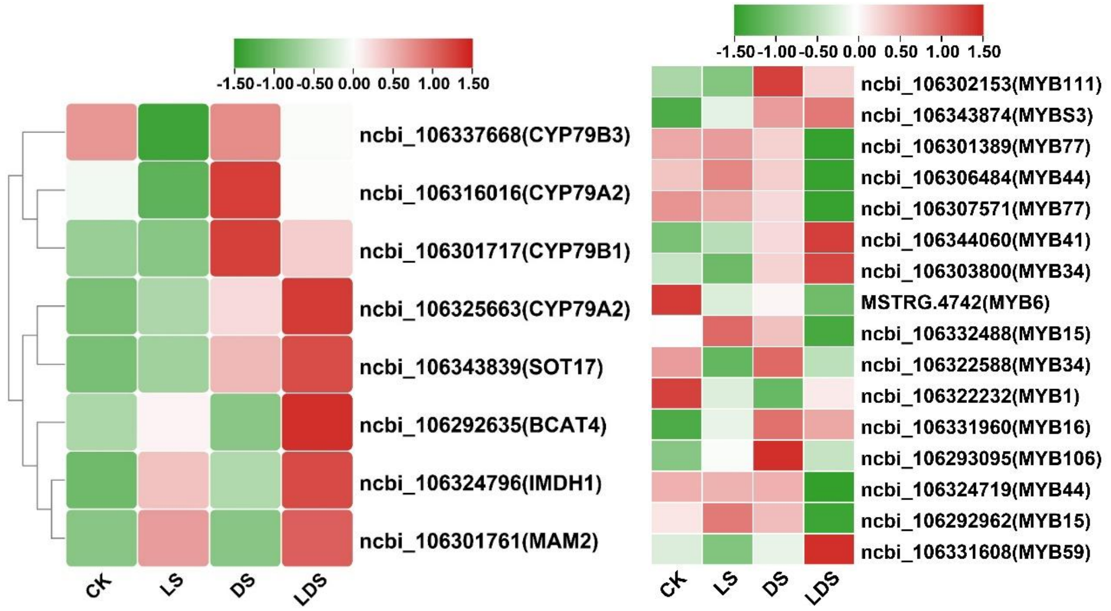

Figure S11. Expression of DEGs related to Glucosinolate biosynthesis pathway and MYB family

[Figure S12]

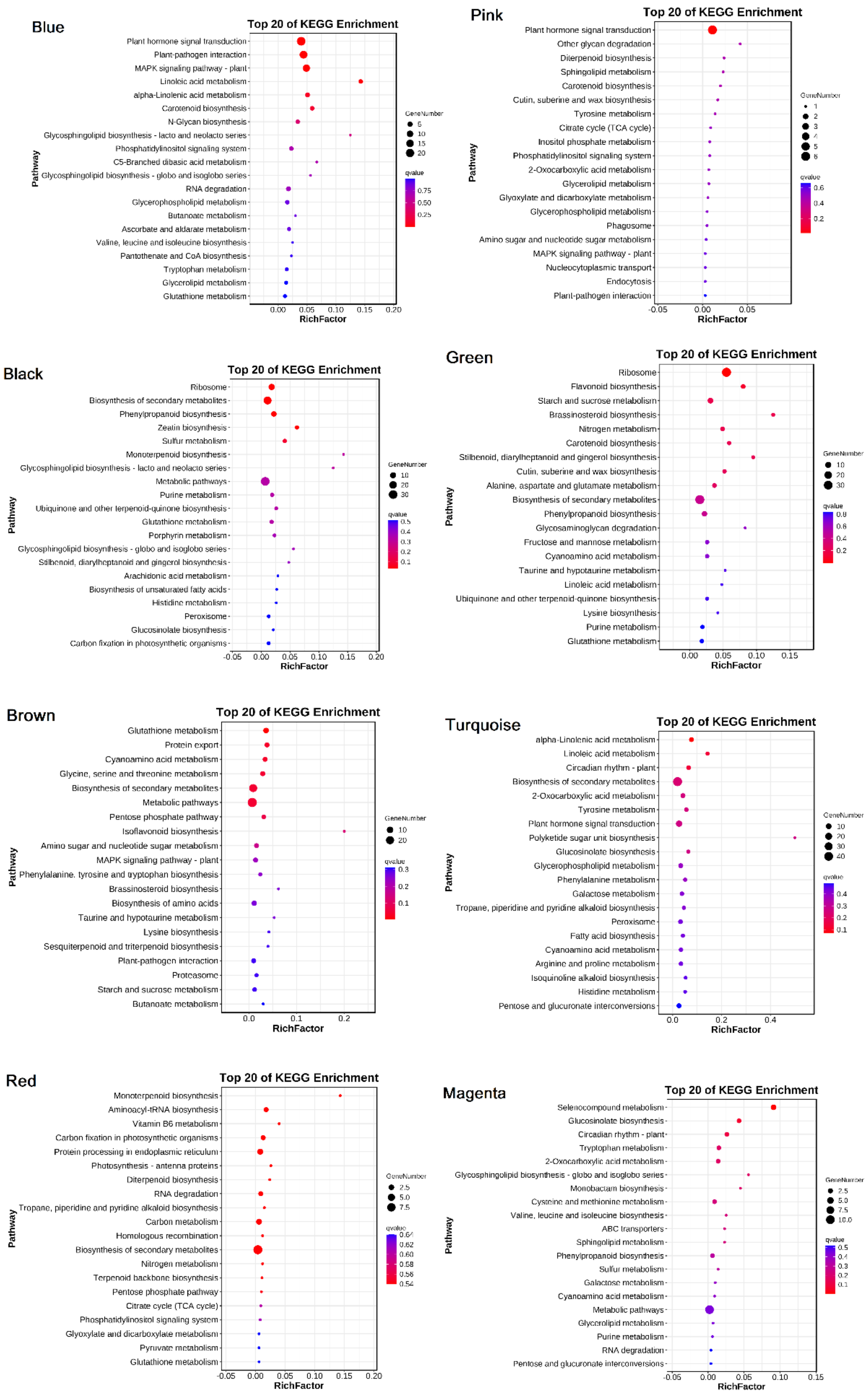

Figure S12. Top 20 KEGG enrichment pathways of 8 modules

[Figure S13]

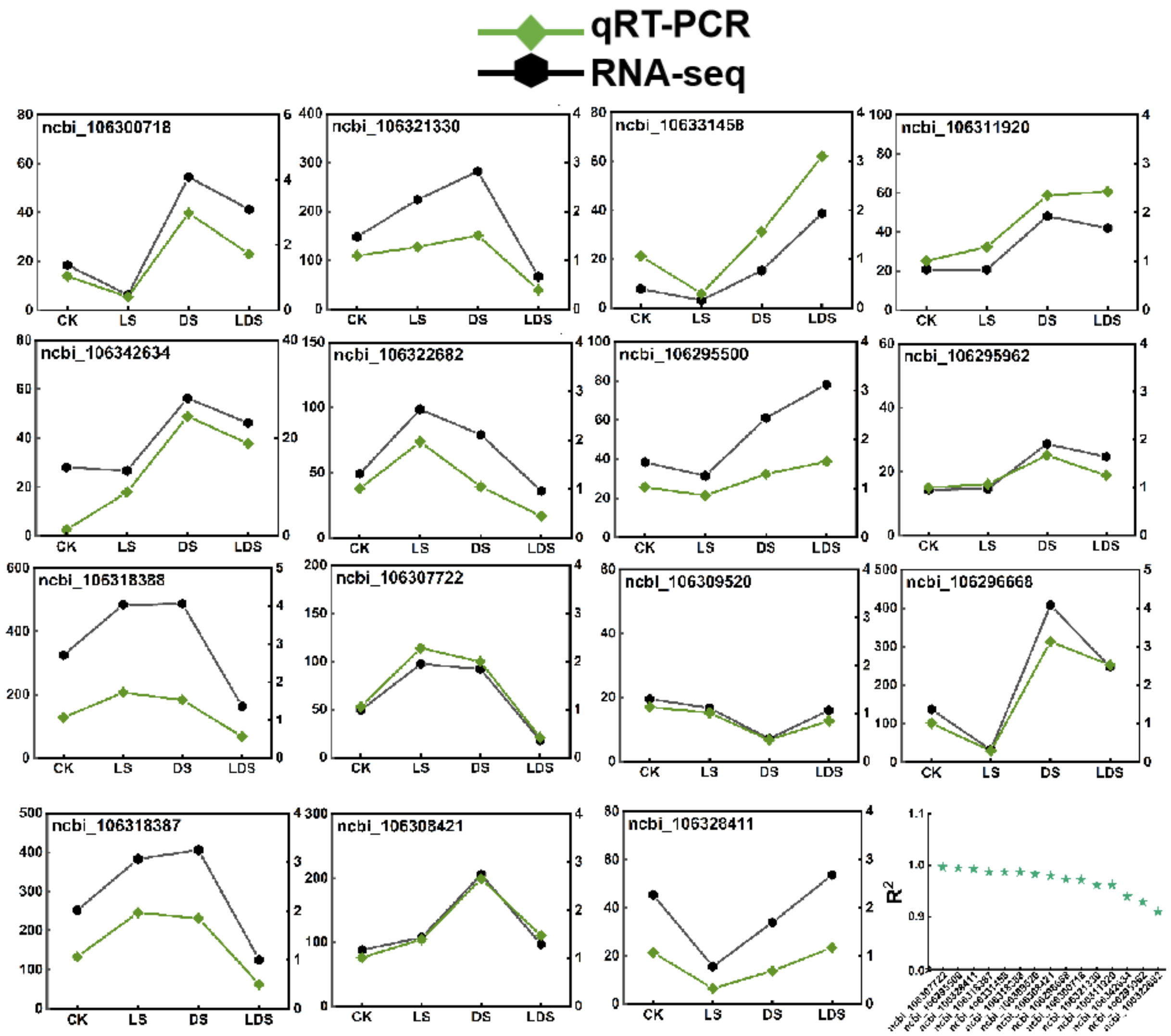

Figure S13. Correlation coefficients between the qRT-PCR and RNA-seq results.

[Figure S14]

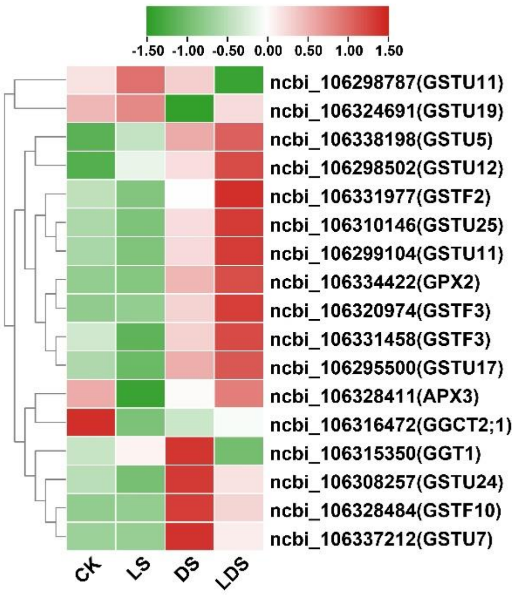

Figure S14. Expression of DEGs related to Glutathione pathway

[Figure 1]

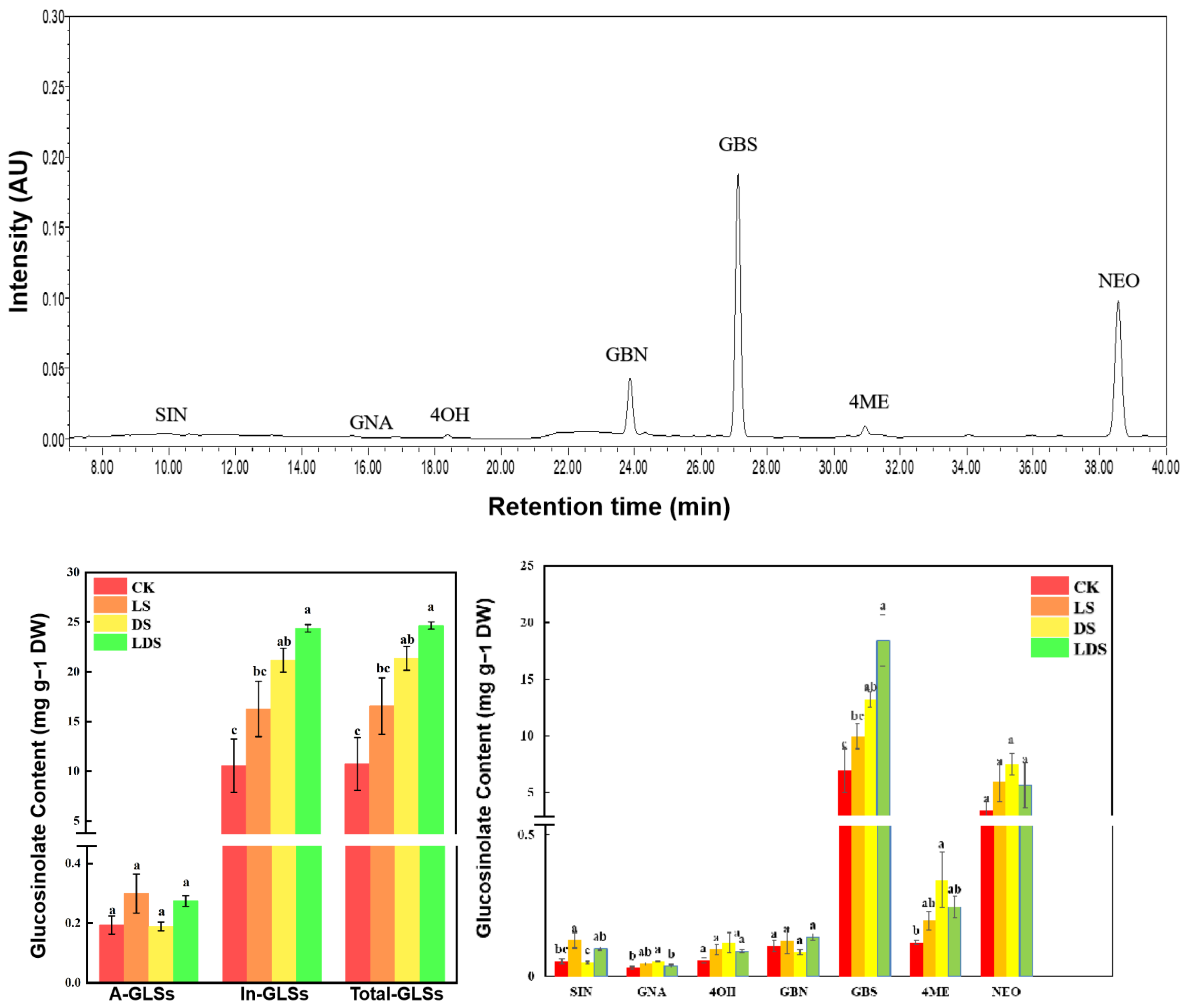

Figure 1. Glucosinolate content of kale under different UV-A treatments. Different lowercase letters reveal significant differences ($p < 0.05$), according to Duncan's test. Vertical bars indicate the standard margin of error. "LS" = Light-UVA Supplementation, "DS" = Dark-UVA Supplementation, "LDS" = Light/Dark-UVA Supplementation. SIN = sinigrin, GNA = gluconapin, 4OH = 4-hydroxyglucobrassicin, GBS = glucobrassicin, GBN = glucobrassicanapin, NEO = neoglucobrassicin, 4ME = 4-methoxyglucobrassicin, In-GLSs = Indolic Glucosinolate, A-GLSs = Aliphatic Glucosinolates..

[Figure 2]

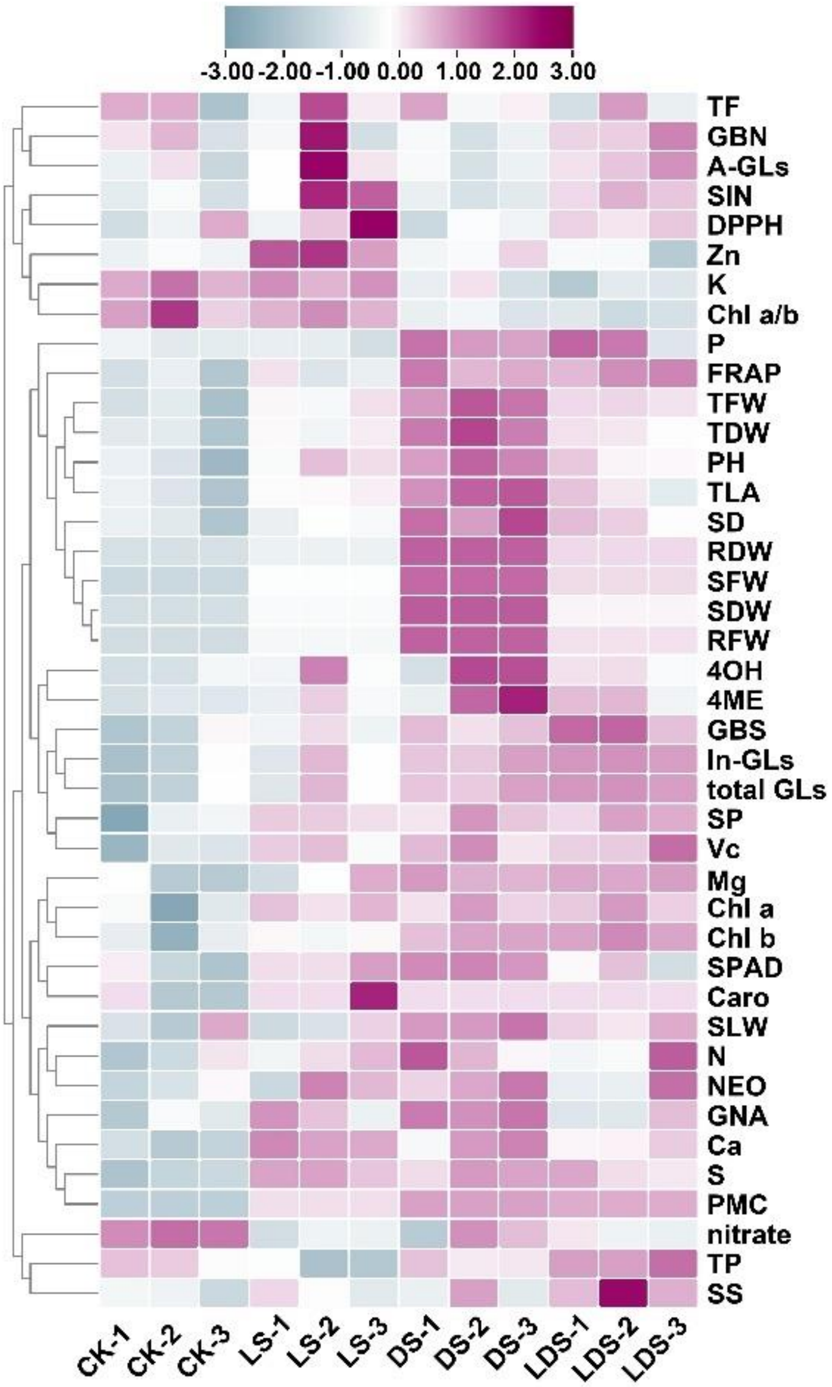

Figure 2. Cluster heatmap analysis summarizing the morphology and quality of kale in supplemental UV-A light treatments and CK. A false-color scale with purple as an increased parameter while blue represents a decreased pa-rameter was using to visualize the results. "LS" = Light-UVA Supplementation, "DS" = Dark-UVA Supplementation, "LDS" = Light/Dark-UVA Supplementation, "PMC" = plant moisture content, "SAW" = specific area weight, "PH" = plant height, "TFW" = total fresh weight, "RDW" = root dry weight, "SD" = stem diameter, "SFW" = shoot fresh weight, "RFW" = root fresh weight, "SDW" = shoot dry weight, "RFW" = root fresh weight, "TDW" = total dry weight, "TLA" = total leaf area, "TP" = total phenolic, "SS" = Soluble sugar, "TF" = total flavonoids, "SP" = soluble protein, "Vc" = Vitamin C, the full name of all GLS variants has been described in Figure 1.

[Figure 3]

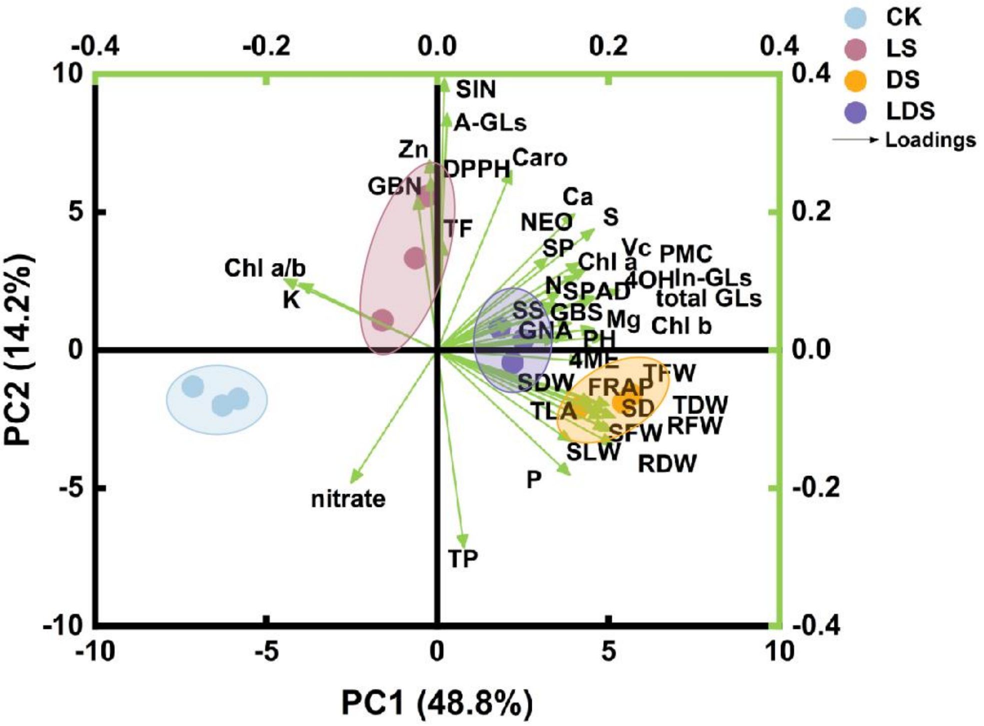

Figure 3. Multivariate principal component analysis presented the relationship between indexes among 4 treatments. The full name has been described in Figure 2. “LS” = Light-UVA Supplementation, “DS” = Dark-UVA Supplementation, “LDS” = Light/Dark-UVA Supplementation

[Figure 4]

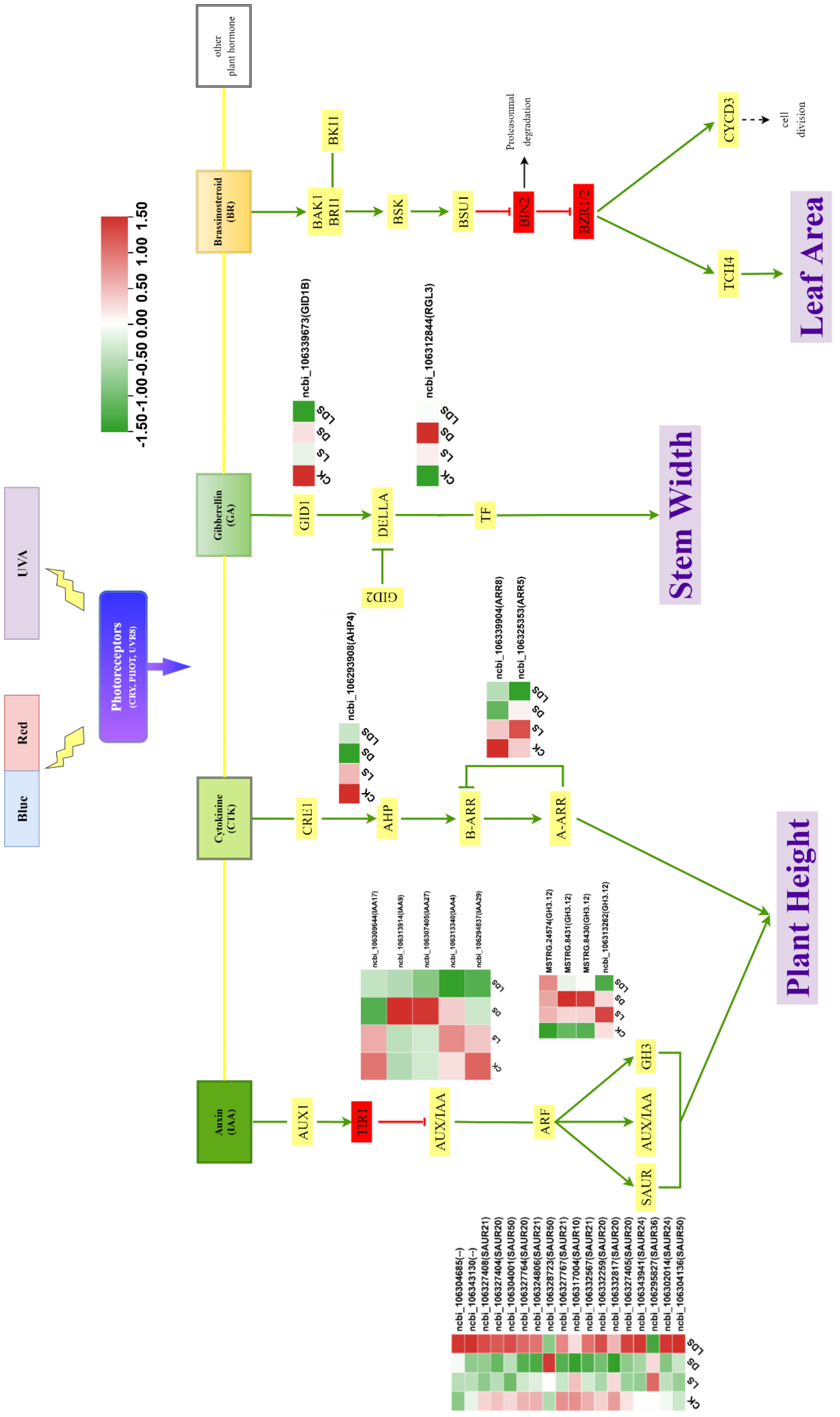

Figure 4. Expression of DEGs on plant hormone signal transduction pathway. Made by diagrams.net. “LS” = Light-UVA Supplementation, “DS” = Dark-UVA Supplementation, “LDS” = Light/Dark-UVA Supplementation

[Figure 5]

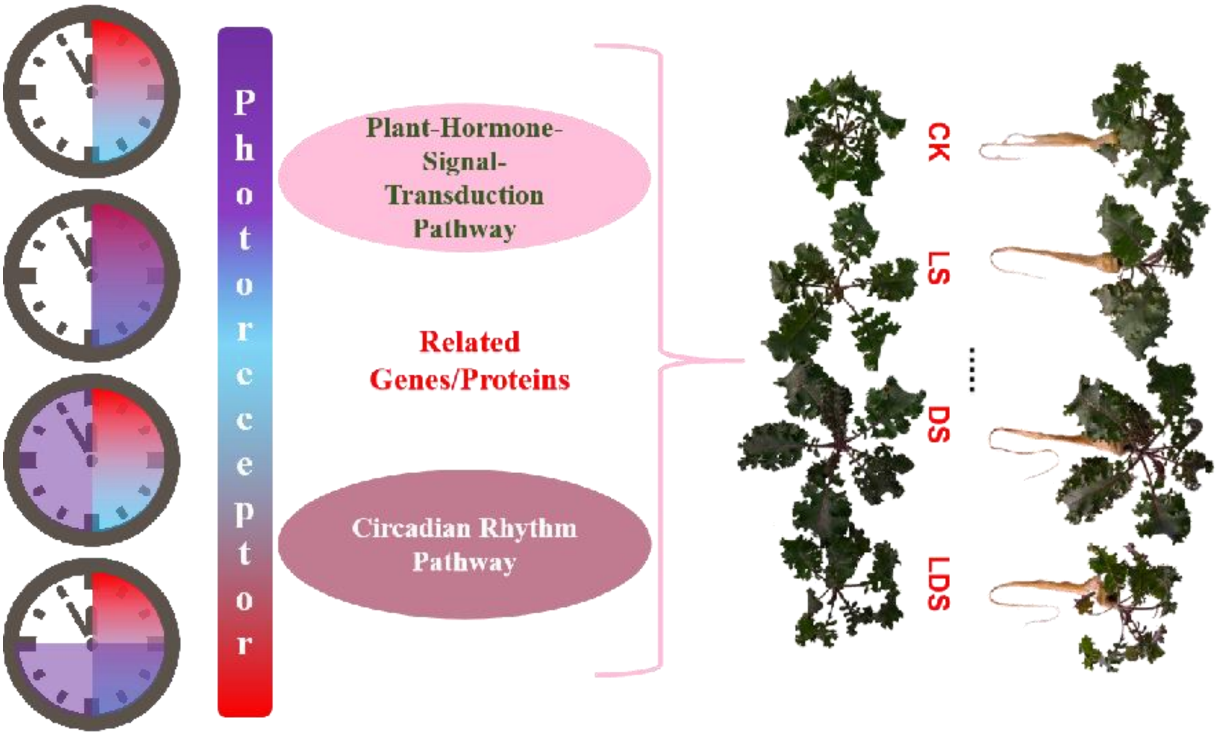

Figure 5. Key regulatory pathways/genes responded to different UV-A supplementation of kale. “LS” = Light-UVA Supplementation, “DS” = Dark-UVA Supplementation, “LDS” = Light/Dark-UVA Supplementation

[Figure 6]

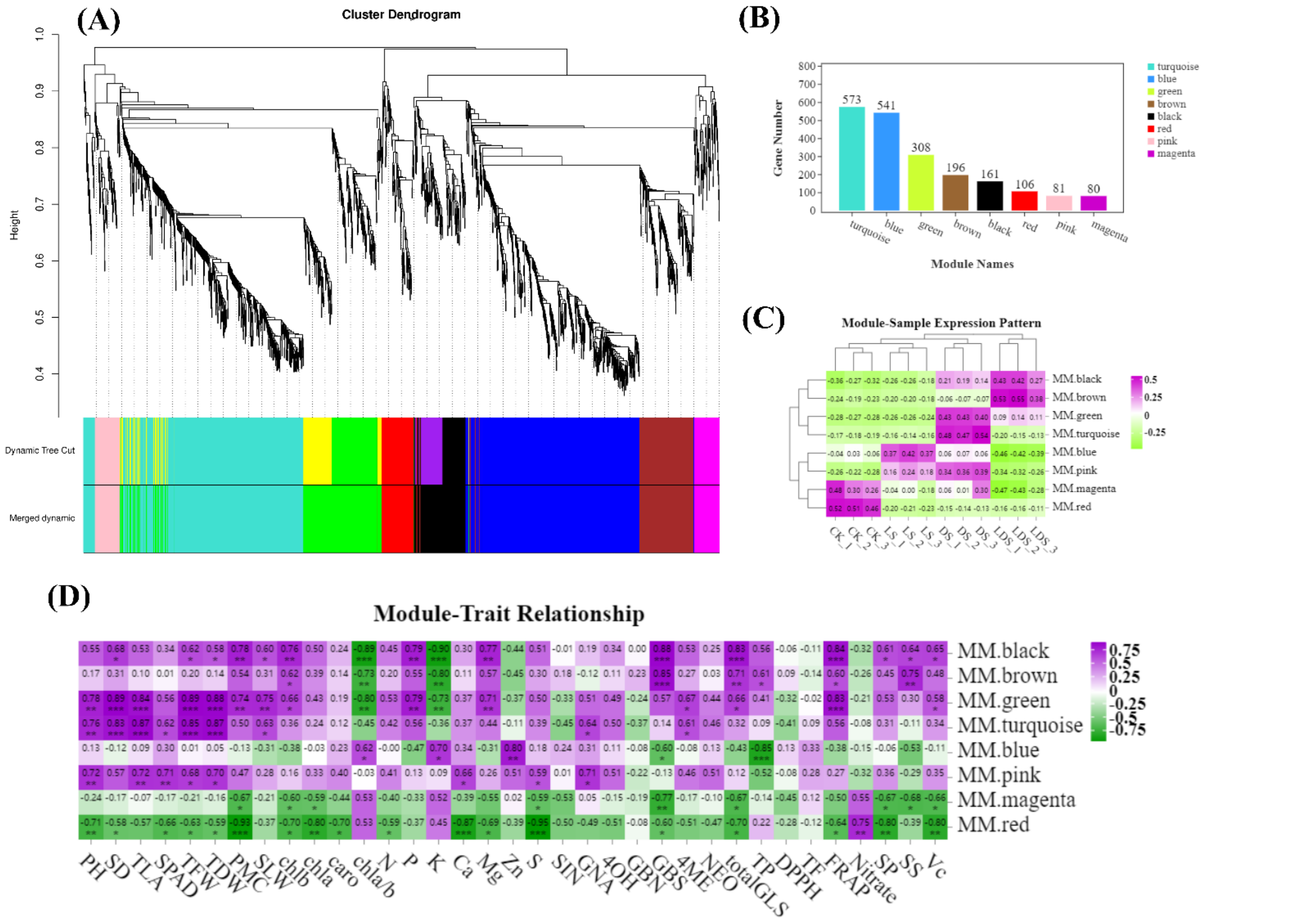

Figure 6. WGCNA of 2047 DEGs. (A) Cluster dendrogram indicating 8 modules of co-expressed genes by WGCNA. (B) Gene numbers of different modules. (C) Module-Sample expression patterns. (D) Module-trait correlations. *p* values were shown as: * $p < 0.05$, ** $p < 0.01$, *** $p < 0.001$. The full name of the traits has been described in Figure 2. The color scale shows correlations from positive (purple) to negative (green). “LS” = Light-UVA Supplementation, “DS” = Dark-UVA Supplementation, “LDS” = Light/Dark-UVA Supplementation
